# Learning and Transfer of Graphomotor Skills in Three 7- to 10-Year-Old Children with Developmental Coordination Disorder: Case Reports

**DOI:** 10.3390/children12121674

**Published:** 2025-12-09

**Authors:** Laureen Josseron, Jérôme Clerc, Caroline Jolly

**Affiliations:** Laboratory of Psychology and NeuroCognition, University Grenoble Alpes, University Savoie Mont Blanc, CNRS, LPNC, 38000 Grenoble, France; laureen.josseron@univ-grenoble-alpes.fr (L.J.); jerome.clerc@univ-grenoble-alpes.fr (J.C.)

**Keywords:** developmental coordination disorder, graphomotor skills, handwriting, motor learning, transfer of learning

## Abstract

**Highlights:**

**What are the main findings?**

- The acquisition of graphomotor abilities is not systematically impaired in children with DCD.
- Graphomotor acquisition depends on the presence and severity of associated dysgraphia.
- Transfer of learning is preserved in children with DCD, whatever the severity of the disorder, and even in the presence of dysgraphia.

**What is the implication of the main finding?**

- Better understanding of the acquisition of fine motor activities and transfer of learning in children with singular DCD.

**Abstract:**

**Background/Objectives:** Children with Developmental Coordination Disorder (DCD) frequently experience handwriting difficulties, or dysgraphia. The association between DCD and dysgraphia has long been observed and described. However, few studies have examined the acquisition and transfer of graphomotor skills in these children, i.e., their ability to learn new graphic gestures and reuse them in new tasks. The objective of this study was to evaluate the acquisition of pseudo-letters and their transfer to different types of tasks in children with DCD. **Methods:** Three case studies of children with DCD, with or without an associated dysgraphia, were compared to an age-matched control group. Participants learned to produce six pseudo-letters during an acquisition phase, then transferred their learning to two tasks: the first assessed the transfer of learned strokes to new pseudo-letters, and the second assessed the transfer of stroke sequences to combinations of two or three pseudo-letters. Performances were analyzed on the basis of four variables: handwritten product quality, and three measures reflecting the handwriting process, i.e., velocity, fluency, and the number of stops during writing. **Results:** Acquisition and transfer abilities differed depending on the presence and severity of dysgraphia. Only the presence of a severe dysgraphia associated with DCD led to a lower quality and a greater on-paper velocity than typically developing children during the learning test. As to transfer, DCD children were able to transfer their learning, even in the presence of a dysgraphia. Only in the case of the second, more distant, transfer task, the presence of a severe dysgraphia led to an increase in velocity and in fluency, and a decrease in the number of stops, in addition to the lower quality. This pattern is typical of handwriting in DCD children with dysgraphia. **Conclusions:** The acquisition of de novo graphomotor skills depends on the presence and severity of a dysgraphia associated with DCD, but not on the severity of other motor impairments. The further transfer of these skills is preserved in DCD children.

## 1. Introduction

According to the DSM-5 [1], the Developmental Coordination Disorder (DCD) is a neurodevelopmental disorder affecting gross and/or fine motor skills [2,3,4]. It affects both the learning and the execution of motor activities in various aspects of everyday life: dressing up, shoelace tying, hair brushing, drawing, scholarly activities, sport, etc. [5,6]. Besides motor deficits, impairments in executive and/or cognitive functions, and perception/action coupling can also be observed, even if less is known about cognitive limits or impairments in DCD children [7,8]. The large diversity of symptoms and deficits observed in DCD makes it a highly heterogeneous disorder [4,5]. In addition, comorbidities are present in most of the children with DCD, in particular learning disorders, including dyslexia, and Attention Deficit with or without Hyperactivity Disorder (ADHD) [9,10,11,12].

The underlying mechanisms of DCD are not clear, and two main hypotheses have been proposed: a deficit in building internal models [13,14], and a deficit in procedural learning [15]. The life quality of children and adults with DCD is clearly impacted, with consequences on many aspects such as, social relationships, self-esteem, increased risk of obesity and cardiovascular diseases, and academic success [6,16,17].

One of the first and major impairments observed in school-aged children with DCD is handwriting difficulties [18,19]. Indeed, handwriting is a complex fine motor task which involves attentional, perceptual, linguistic, and motor skills (for a review see [20]). Handwriting acquisition occupies a large proportion of school activities, since its mastery is necessary for the further acquisition of higher-level skills such as grammar, syntax, or text composition [20]. Handwriting deficits, also known as dysgraphia, are observed in 50 to 88% of children with DCD according to different authors [21,22]. It can affect both the handwriting process through changes in handwriting speed, pauses, and in-air time, and the handwriting product by decreasing the quality of handwriting. Analyzing these two dimensions (i.e., the product and the process) when studying handwriting is important and necessary to precisely identify the specific difficulties of each child. Since dysgraphia can impact other types of learning and eventually lead to academic failures [23], it is thus crucial to unravel the mechanisms leading to dysgraphia in DCD children, in order to help them to cope with this issue.

Another skill which plays an essential role in the efficacy of learnings at school is transfer of learning, which is defined as the capacity to adapt prior learning to new tasks and/or situations [24,25,26]. Transfer of learning concerns both cognitive and motor aspects. While handwriting acquisition and deficits in DCD children are increasingly better understood, the transfer of handwriting in DCD children remains underexplored, despite its importance for the efficacy of interventions. Transfer of learning can be assessed after an initial learning phase by presenting new tasks to participants. These new tasks may differ from the initial learning task through modifications of the task itself, the context, or the time interval between the presentation of the initial task and the transfer task [27]. Thus, transfer tasks can be more or less distant from the initial learning depending on variations along these different dimensions: the greater the distance between the transfer task and the initial learning task, the farther the transfer. Transfer can therefore be conceived as a continuum ranging from near to far transfer, depending on their distance from the initial learning task. Recently, Josseron et al. [28] reviewed the literature in regard to transfer of motor learning abilities in children with DCD. They reported that these children have more difficulties than Typically Developing (TD) children with transferring motor learning when the transfer task is more distant from the trained task, but not when the two tasks remain close, which is in line with recent literature on transfer in non-DCD individuals showing the relative easiness of transfer when the two tasks are close on the near-to-far continuum [27,29,30,31,32,33]. Among the articles included in this scoping review, only one deals with handwriting [34]. These authors proposed a graphomotor training task consisting in copying a pseudo-letter with guiding dots, followed by a transfer task in which the guiding dots were removed. They found that DCD children learn to write the pseudo-letter as efficiently as their TD peers, but display weaker transfer performances. The authors explain this greater difficulty in transfer among children with DCD by the fact that the transfer task is more distant from the initial learning (referring to it as “far transfer”), which is attributed to a larger difference in spatial constraints between the learning and transfer tasks. In a recent study, we investigated de novo learning and transfer of graphomotor skills in TD children aged 7 to 10 years old and in adults, using a task consisting of copying several pseudo-letters of increasing complexity and with various constraints (Josseron et al., submitted). Both the product and the process were analyzed. We found that by the age of 7, children reached adult-like performances during the learning phase but struggled to transfer these skills, as attested by the decrease in quality, particularly when the transfer task was complex and more distant from the initial learning task. In 8- and 9-year-old children, quality was maintained in the two transfer tasks but at the cost of kinematic adjustments (i.e., reduced fluency and increased number of stops), while 10-year-old children performed similarly to adults in all tasks.

In the present article, we were interested in investigating the transfer of graphomotor skills in children with DCD without other known neurodevelopmental disorders, with the same paradigm as Josseron and colleagues (submitted). Since recruiting a large sample of children with singular DCD was really challenging because of the scarcity of such a specific clinical profile, and because of the wide heterogeneity of the DCD profiles as mentioned above [4,5], we chose to study the case of three DCD children aged 7.7 to 10.8 years old. These children presented different profiles, which were particularly interesting in regard to our research question. The first was a child with only a moderate DCD and no dysgraphia, the second presented a severe DCD and a mild dysgraphia, and the third one presented a mild DCD and a severe dysgraphia. These different clinical profiles allowed us to address two questions regarding learning and transfer of graphomotor skills: (i) are they impacted by the severity of the DCD? and (ii) are they impacted by the presence and severity of an associated dysgraphia? We thus proposed to the children the same pseudo-letter copying task, on a sheet of paper affixed onto a graphic tablet, allowing access both to the handwriting product (i.e., quality of the pseudo-letters produced) and to the process through kinematic variables. The quality and process of the handwritten productions were assessed at each phase of the experiment, and we compared the results obtained by each DCD child to an age-matched control group. Following the study by Adi-Japha & Brestel [34], we hypothesized that children with DCD would demonstrate similar skills to TD children when learning pseudo-letters, but that their performance in the transfer tasks would be lower than that of their peers. This should be reflected in weaker outcomes for both the product and the process of their written productions.

## 2. Materials and Methods

### 2.1. Participants

Fifty-seven TD children aged 7 to 10 years old were included in the study. Their demographic characteristics are presented in Table 1. They constituted the three control groups according to grade. All children completed the French version of the BHK test [35] to ensure the absence of dysgraphia, and three items corresponding to the three components of the MABC-2 test [36] to ensure the absence of DCD [37,38]: placing pegs with the preferred hand (Manual Dexterity), throwing beanbag onto mat (Aiming and Catching) and walking heel-to-toe-forwards (Balance).

Three children with DCD were recruited through advertisements disseminated by professionals. Their characteristics are presented in Table 2. The MABC-2 test confirmed the presence of a DCD in the three children. Handwriting was also evaluated using the BHK test [35]. Two children (case DCD2 and case DCD3) presented a dysgraphia as attested by their speed and/or quality scores. The absence of comorbidity with other neurodevelopmental disorders, the absence of neurological impairment, and the absence of uncorrected visual impairment were confirmed by the children’s parents.

This study was approved by the University ethics committee (CERGA-Avis-2022-9). Written consent from all parents and oral consent from all children were acquired before the experiment.

### 2.2. Materials

**Figure 1:**
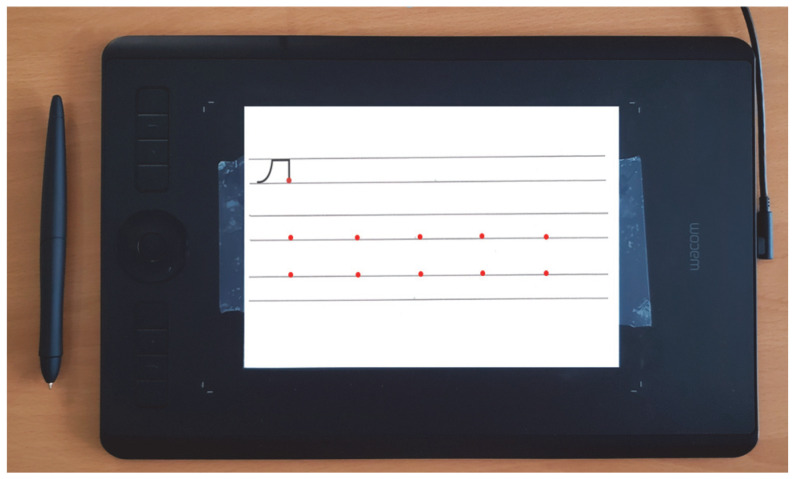
Graphic tablet with the paper sheet affixed, and the ink pen (left). The model of the pseudo-letter is shown on top. In this example, the children had to copy the item ten times below the model, from right to left, starting with the red dots, between the two guiding lines (acquisition phase).

We used the exact same material as described in Josseron et al. (submitted). Briefly, we designed 12 pseudo-letters, each composed of three to six strokes. The pseudo-letters were presented on a sheet of paper between 2 guiding lines spaced 1 cm apart, and affixed on a Wacom© Intuos Pro tablet (Figure 1). All tasks were performed using the Wacom© ink pen to ensure ecological conditions.

### 2.3. Procedure

Again, the procedure was the same as described in Josseron et al. (submitted). In the acquisition phase, the children were asked to copy six pseudo-letters individually, ten times each, between the guiding lines and from right to left, starting from a red dot (see Figure 1 for an example). In the first transfer task, children were then asked to copy three new pseudo-letters composed of strokes already learned in the acquisition phase, with the same instructions as before. They first copied the pseudo-letters twice without constraint (Transfer 1 Session 1), then again twice with the constraint of not lifting the pen (Transfer 1 Session 2). A second transfer task of higher complexity was then proposed, in which the three items to copy were pseudo-syllables composed of two or three of the pseudo-letters learned in the acquisition phase. These combinations of pseudo-letters led to the creation of a new stroke that was not present in the isolated pseudo-letters. Children had to copy the items twice without additional instruction (Transfer 2 Session 1), and then with the constraint of not lifting the pen (Transfer 2 Session 2).

### 2.4. Measures

Four measures were evaluated. First, the quality of the handwritten production was evaluated with an 11- or 12-point scale (for the pseudo-letters and the combinations of 2 or 3 pseudo-letters, respectively), as described in Josseron et al. (submitted). The pseudo-letter quality scale is presented in Appendix A, Table A1. The 11 criteria were: global similarity to the model, one end touching the red dot, no interrupted connection, starting from the red dot, correct proportion between the 2 guiding lines, no retouching or erasure, no exceeding stroke, respected angles, respected straight lines, respected curves; and the additional criterion was new segment quality. Each item was scored 0 if the criterion was not present, and 1 or 2 (for the “global similarity to the model” criterion) if present. Each handwritten production thus obtained a maximal score of 11 or 12. In addition, the handwriting process was evaluated through 3 kinematic features known to discriminate dysgraphic handwriting, and extracted using the Wacom© tablet and a dedicated software [39]. These features were: the mean on-paper velocity which measures the velocity of the pen on the paper (in cm/sec) [40,41], the number of abnormal stops during writing (when the pen pauses on the paper for periods longer than 35 milliseconds) [42], and the writing fluency through the SNvpd (Signal-to-Noise velocity peak difference) [43]. This feature represents the number of abnormal velocity peaks, reflective of abnormal micro-fluctuations of velocity during tracing [43]. The higher the SNvpd, the lower the fluency.

### 2.5. Statistical Analyses

We performed 2 types of analyses. We first analyzed the raw scores for the four measures described above to compare the performances of each DCD child with the peer control group. We next performed a second analysis aiming at investigating the mechanisms underlying transfer performances, by considering the changes in performances from the learning test to the transfer tasks. To that purpose, for each child we subtracted (for quality, SNvpd and number of stops) or divided by (for velocity) the values obtained during the learning test from the values obtained during each of the transfer phases. This allowed for control of interindividual differences during the acquisition phase, thus obtaining a purer measure of transfer performances with each child acting as its own control [44].

For both analyses, the comparison between the values obtained by each DCD child was then compared to the age-peered control group using the *SingleBayes* program, which implements Bayesian methods for comparison of a single case’s score to scores obtained in a control sample. The *SingleBayes* program provides a *p*-value, an effect size (Z_cc_, for Case–Controls), and a 95% confidence interval for the effect size [45].

## 3. Results

### 3.1. Case DCD1

**Figure 2:**
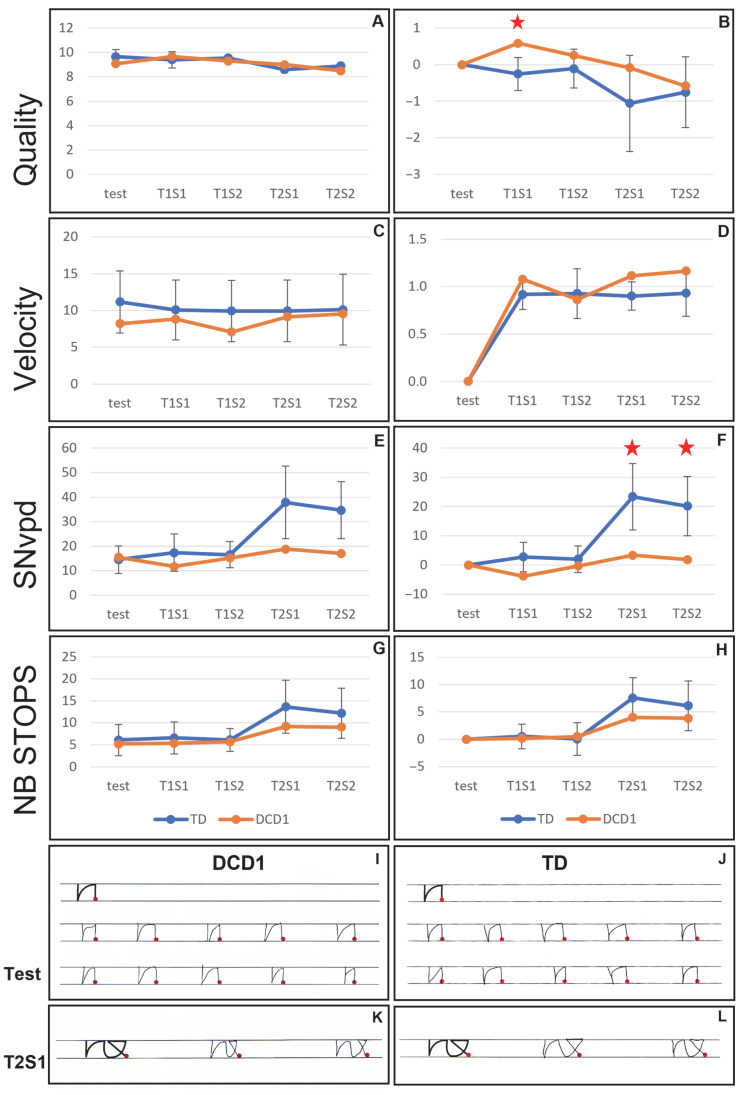
(**A**–**H**) Results of the comparison of the performances of the DCD1 child (orange) and the 7-year-old control group (blue) during each phase of the experiment. The comparisons of the raw performances (left panels) and of the differences between the test and the transfer phases (right panels) are displayed for quality (**A**,**B**), velocity (**C**,**D**), SNvpd (**E**,**F**), and number of abnormal stops (**G**,**H**). (**I**–**L**) Examples of the productions of the DCD1 child during the learning test phase (**I**) and during the T2S1 phase (**K**), and examples of the same pseudo-letters produced by a 7-year-old typically developing child during the learning test phase (**J**) and the T2S1 phase (**L**). Test: learning test phase. T1S1: session 1 of transfer task 1. T1S2: session 2 of transfer task 1. T2S1: session 1 of transfer task 2. T2S2: session 2 of transfer task 2. The red stars indicate the significant differences between the DCD child and the control group.

The DCD1 child is a 2nd-grade boy, aged 7.75 years old, presenting a mild DCD (16th percentile) and no dysgraphia. Results of the comparison between the DCD1 child’s performances and the 7-year-old control group are presented in Figure 2. The complete dataset is given in Appendix A, Table A2.

When first analyzing the raw performances obtained at each phase of the experiment, no difference between the DCD child and the control group was observed, for none of the four parameters (*p* > 0.05; Figure 2, panels A, C, E, and G). When analyzing the differences between the learning test and the transfer phases, a few significant differences were observed between the DCD child and the control group. Indeed, the quality increased for the DCD child during session 1 of the transfer task 1 (T1S1) while it decreased for the control group (*p* = 0.05; Figure 2, panel B). Likewise, the DCD child displayed a significantly lower increase in SNvpd (i.e., a lower decrease in fluency) during sessions 1 and 2 of the transfer task 2 (T2S1 and T2S2) than the control group (*p* = 0.05; Figure 2, panel F). In contrast, no difference between the DCD child and the control group was observed for the velocity and the number of stops (*p* > 0.05; Figure 2, panels D and H).

Examples of the handwritten productions of the DCD1 child and an age-peered TD child are shown in Figure 2, panels I to L. The productions of the two children look similar, in agreement with the statistical analyses.

### 3.2. Case DCD2

**Figure 3:**
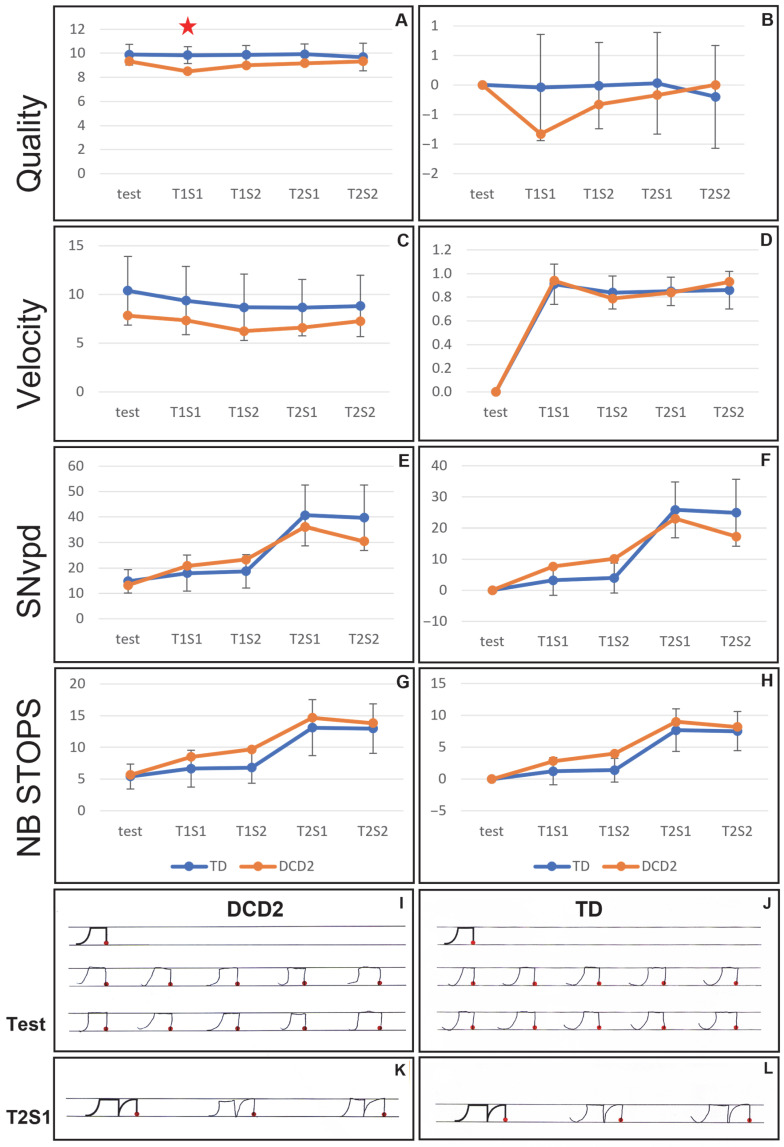
(**A**–**H**) Results of the comparison of the performances of the DCD2 child (orange) and the 9-year-old control group (blue). The comparisons of the raw performances (left panels) and of the differences between the test and the transfer phases (right panels) are displayed for quality (**A**,**B**), velocity (**C**,**D**), SNvpd (**E**,**F**), and number of abnormal stops (**G**,**H**). (**I**–**L**) Examples of the productions of the DCD2 child during the learning test phase (**I**) and during the T2S1 phase (**K**), and examples of the same pseudo-letters produced by a 9-year-old typically developing child during the learning test phase (**J**) and the T2S1 phase (**L**). Test: learning test phase. T1S1: session 1 of transfer task 1. T1S2: session 2 of transfer task 1. T2S1: session 1 of transfer task 2. T2S2: session 2 of transfer task 2. The red star indicates the significant differences between the DCD child and the control group.

The DCD2 child is a 4th-grade boy, aged 9.33 years old, presenting a severe DCD (0.1st percentile) and a mild dysgraphia. Results of the comparison between the DCD2 child’s performances and the 9-year-old control group are presented in Figure 3. The complete dataset is given in Appendix A Table A3.

When first analyzing the raw performances obtained at each phase of the experiment, the only significant difference between the DCD child and the control group was for the quality, with a decrease during the T1S1 phase for the DCD2 child compared to the control group (*p* = 0.04; Figure 3, panel A). No difference was observed for the velocity, SNvpd and number of stops (*p* > 0.05; Figure 3, panels C, E and G). When analyzing the differences between the learning test and the transfer phases, no significant difference between the DCD child and the control group was observed (all *p* > 0.05; Figure 3, panels B, D, and H).

Examples of the handwritten productions of the DCD2 child and an age-peered TD child are shown in Figure 3, panels I to L. The productions of the two children look similar, in agreement with the statistical analyses.

### 3.3. Case DCD3

**Figure 4:**
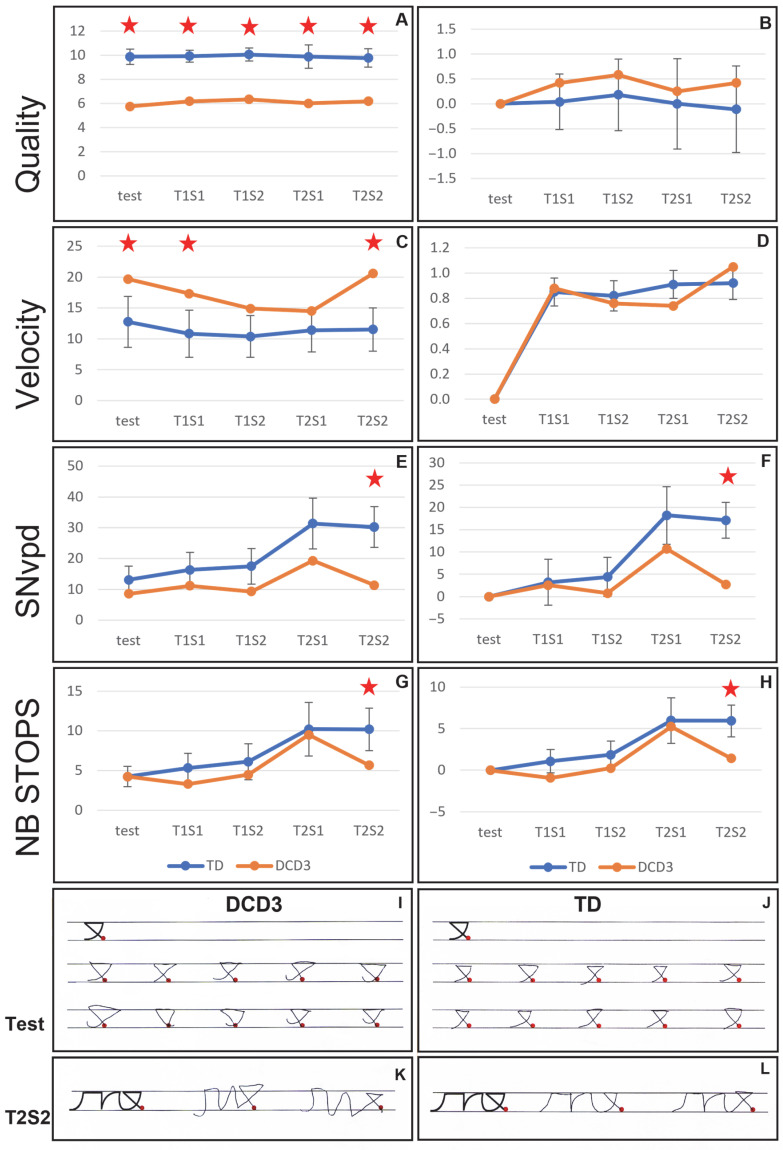
(**A**–**H**) Results of the comparison of the performances of the DCD3 child (orange) and the 10-year-old control group (blue) during each phase of the experiment. The comparisons of the raw performances (left panels) and of the differences between the test and the transfer phases (right panels) are displayed for quality (**A**,**B**), velocity (**C**,**D**), SNvpd (**E**,**F**), and number of abnormal stops (**G**,**H**). (**I**–**L**) Examples of the productions of the DCD3 child during the learning test phase (**I**) and during the T2S1 phase (**K**), and examples of the same pseudo-letters produced by a 10-year-old typically developing child during the learning test phase (**J**) and the T2S1 phase (**L**). Test: learning test phase. T1S1: session 1 of transfer task 1. T1S2: session 2 of transfer task 1. T2S1: session 1 of transfer task 2. T2S2: session 2 of transfer task 2. The red stars indicate the significant differences between the DCD child and the control group.

The DCD3 child is a 5th-grade girl, aged 9.33 years old, presenting a moderate DCD (9th percentile) and a severe dysgraphia affecting primarily handwriting quality. Results of the comparison between the DCD3 child’s performances and the 9-year-old control group are presented in Figure 4. The complete dataset is given in Appendix A Table A4.

When first analyzing the raw performances obtained at each phase of the experiment, major differences between the DCD child and the control group appeared for the four parameters. First, the quality of the DCD3 child productions was significantly lower during all phases of the experiment (all *p* < 0.001; Figure 4, panel A). Moreover, the velocity was significantly higher for the DCD child during the learning test phase (*p* = 0.05), session 1 of transfer task 1 (T1S1; *p* = 0.05) and session 2 of transfer task 2 (T2S2; *p* = 0.007) (Figure 4, panel C), and the SNvpd was significantly lower for the DCD child during the T2S2 phase (*p* = 0.005; Figure 4, panel E). Finally, the number of abnormal stops was significantly lower for the DCD child during the T2S2 phase (*p* = 0.05; Figure 4, panel G).

When analyzing the differences between the learning test and the transfer phases, no difference between the DCD child and the control group was observed for the quality and the velocity (*p* > 0.05). In contrast, a significantly higher decrease in SNvpd (i.e., a higher increase in fluency) was observed during the T2S2 phase for the DCD child compared to the control group (*p* = 0.001; Figure 4, panel F). Moreover, a significant decrease in the number of stops during the T2S2 phase was observed for the DCD child compared to the control group ((*p* = 0.016); Figure 4, panel H).

Examples of the handwritten productions of the DCD3 child and an age-peered TD child are shown in Figure 4, panels I to L. The lower quality of the DCD3 child productions is in agreement with the statistical results.

## 4. Discussion

The aim of this study was to evaluate graphomotor learning and transfer of pseudo-letters in three children with singular DCD compared to age-matched TD children, as a means to better understand the impact of DCD on handwriting acquisition. We created an acquisition–transfer paradigm inspired by the study of Adi-Japha & Brestel [34] and using pseudo-letters in order to assess the development of de novo graphomotor learning. Through three case studies with different clinical profiles, we evaluated participants’ written production during the acquisition and transfer phases using a quality scale, and their writing processes by analyzing three kinematic variables: velocity [40,41], fluency [43], and the number of abnormal stops [42].

The three DCD cases presented here were particularly interesting in regard to our research interests. Indeed, these three children differed in the severity of the disorder and in the presence or not of an associated dysgraphia: the DCD1 case presented only a moderate DCD, the DCD2 case presented a severe DCD with a mild dysgraphia, while the DCD3 case presented a moderate DCD but a severe dysgraphia. These differences allowed us to investigate the impact of the severity of motor impairments and of the presence and severity of an associated dysgraphia on graphomotor learning and transfer. We found that the performances during the learning test varied among children with DCD. While the DCD1 and DCD2 children were as good as their TD peers when learning pseudo-letters, the DCD3 child obtained lower quality scores than the control group, in addition to a higher writing speed during both the learning test and two of the four transfer sessions. This is a well-documented characteristic of handwriting processes in children with DCD and dysgraphia, who frequently exhibit higher instantaneous pen-on-paper velocity, reflecting a difficulty in precisely controlling graphic movements and resulting in a lower quality in graphic productions [40,41,46,47]. On the contrary, this pattern was observed neither in the DCD1 child (no dysgraphia) nor in the DCD2 child, who presented a very severe DCD but only a moderate dysgraphia. Thus, the presence of severe motor difficulties in the DCD2 child did not prevent the motor learning of pseudo-letters. Our results thus suggest that only the presence and severity of an associated dysgraphia affected the handwritten productions both at the quality and the process level in the DCD3 child, independently of the presence and severity of the other motor impairments. This suggests that handwriting may imply specific motor mechanisms which, when affected in children with DCD and dysgraphia, result in specific impairments (i.e., lower quality, lower precision of fine movements, and increased on-paper velocity) [40,41,47]. This hypothesis is in agreement with previous observations showing distinct impairments when dysgraphia is associated with developmental dyslexia or with DCD [47,48,49]. Furthermore, the DCD3 child obtained lower performances during all phases of the experiment, but their progression throughout the experiment parallels that of TD children. This observation supports previous results showing that for each child with a combined DCD and dysgraphia, handwriting performances reach a steady-state level reflecting his/her own maximal skills [41]. This is exactly what we observed for the DCD3 case, whose maximal quality score was around 6 for all phases of the experiment, whereas it was around 10 for the control group. This result thus supports the hypothesis that even with training, this child was not able to go beyond a certain level and reach the level of TD children.

In this study, the first transfer task involved applying the strokes learned during the acquisition phase to draw new pseudo-letters. When children with DCD were asked to trace the pseudo-letters without any constraint (Session 1), only those with an associated dysgraphia, whatever its severity (DCD2 and DCD3 children), experienced more difficulty in completing the task. However, these two children did not experience greater difficulty than their peers in transferring from the learning test to this first transfer task, despite overall lower raw performance: the evolution of their performances throughout the experiment parallels that of TD children This was particularly true for the DCD3 child, who showed higher velocity values than the TD children during this task, as was the case for the learning phase as described above. Moreover, in the second session of the first transfer task, we expected that adding the constraint of not lifting the pen would increase the task’s difficulty. However, only the DCD3 child showed a lower overall quality compared to their TD peers, but quality was not different from their initial level, without exhibiting notable differences in the writing process. This suggests that, despite the introduction of a new constraint (e.g., not lifting the pen), DCD children were able to transfer their learning, even in the presence of severe global motor impairments (case DCD2) or severe dysgraphia (case DCD3). This observation is in contradiction with previous results obtained by Adi-Japha & Brestel [34]. This discrepancy may be due to the way our experimental protocol was built. Indeed, prior exposure to an unconstrained session may have facilitated the completion of this second constrained session. In addition, the relative simplicity of this first transfer task, which is composed of only three strokes, combined with an initial experience without any constraint during the acquisition phase, may have helped the further execution of the same task with the constraint of not lifting the pen. Interestingly, this advantage of session 2 over session 1 during the first transfer task is also observed in the second, more difficult, transfer task involving the production of combinations of pseudo-letters. Especially for the DCD3 case, quality remained lower than that of the TD group but did not decrease during the transfer task 2 session 2, compared to her initial performances. At the procedural level, this child showed a marked increase in writing velocity, a greater writing fluency, and a decrease in the number of stops during session 2. These observations support the hypothesis of an advantage of session 2 over session 1 due to a bias in our protocol, which may have facilitated the transfer under the constraint of not lifting the pen.

The assessment of quality during the learning test and both transfer tasks allowed us to evaluate the acquisition and transfer of graphomotor skills in all children. From a theoretical point of view, the results of our case studies first suggest that DCD children are able to reach a certain level of graphomotor skills, depending on the presence and severity of an associated dysgraphia. Only the presence of a severe dysgraphia seems to impact graphomotor learning, leading to lower production quality and specific kinematic features such as higher on-velocity and greater fluency, which are well-known characteristics of the writing processes in children with DCD and dysgraphia [41,47]. Second, the evolution of the performances of DCD participants throughout the experiment did not differ from that of their peers, even for the DCD3 child who displayed lower but stable quality performances (Figure 4 panels A and B). This observation suggests that children with DCD are able to transfer graphomotor learning to the same extent as TD children, even in the presence of severe global motor impairments (case DCD2) or severe dysgraphia (case DCD3). This observation contradicts our initial research hypotheses and previous results obtained by Adi-Japha & Brestel [34] and others (reviewed in [28]) who showed that children with DCD struggle to transfer motor skills. A possible explanation for this discrepancy is that we only present three case reports, which may not be representative of all DCD children. Another possible explanation is that we focused here on a very specific fine motor task, i.e., handwriting. Possibly, handwriting may present some specificities distinct from other motor tasks, which could be easier to transfer.

The observation that transfer of graphomotor skills is preserved even in the presence of severe global motor impairments (case DCD2) or severe handwriting deficits (case DCD3) is interesting in regard to the existing theoretical models of motor learning in DCD. Two main hypotheses have been proposed: a deficit in building internal models [13,14], and a deficit in procedural learning [15]. The first hypothesis, which is the most widely accepted among specialists, suggests a difficulty in building, adapting, and/or using internal models. Anticipation and adaptation skills are important in motor actions, and a deficit in internal models may particularly affect these skills in DCD children. Since transfer requires adaptation [26,29], our results thus suggest that adaptation seems to be preserved in our task, since all three DCD children were able to adapt and succeed in two new and more difficult tasks. In addition, the fact that the DCD1 and DCD2 cases obtained performances comparable to those of the control group supports the idea that these children were able to build efficient internal models for this task. Only the DCD3 case, who has a severe dysgraphia, may not have been able to build internal models strong enough to reach performances comparable to TD children. Altogether, these results thus support the hypothesis of an internal model deficit in DCD when a dysgraphia is associated. This is in line with recent results showing a preserved ability to build internal models in the case of singular DCD, but not in the presence of comorbidities [50,51]. The second explanatory hypothesis for motor impairments in DCD suggests a deficit in procedural learning, i.e., a difficulty in learning sequences of movements [15]. Since our protocol did not directly test procedural learning, we cannot draw any conclusion regarding this point. However, the lower quality of the handwritten production of the DCD3 case, who has a severe dysgraphia, together with her increased velocity and fluency, suggests the existence of particular handwriting processes in this child, which may relate to deficits in procedural learning. Increased writing velocity and fluency, although detrimental to quality, may represent adaptive mechanisms to succeed in a difficult task [52,53].

Another interesting observation is that the second session of the second transfer task, which included both more complex strokes and the constraint of not lifting the pen, can be considered as a more distant transfer task compared to the previous task and sessions [27,54]. Thus, in this study, even though the transfer tasks remain close to the initial learning task, our results suggest that transfer of graphomotor learning can be preserved in children with DCD, even if the transfer tasks are farther from the initial learning task on the near-to-far transfer continuum.

Beyond motor acquisition, the transfer of motor skills is considered a key component of motor learning [55]. In this study, the acquisition–transfer paradigm made it possible to assess not only the initial component of learning but also its transfer, something that is rarely performed in children with DCD [28]. Although we did not measure retention because of the impossibility of meeting the children later on, our paradigm still allowed a detailed analysis of their learning difficulties. Assessing both the learning of pseudo-letters and their transfer highlighted differences in handwritten products and handwriting processes across the stages of learning. Although we are presenting here only three case reports, comparisons between DCD and TD children and across DCD children led to new insights into the understanding of motor skills in DCD children. Future studies in children with DCD should thus consider transfer of learning more systematically. In addition, we present here a completely new experimental procedure that can be useful to researchers and clinicians interested in the transfer of learning.

Our study, however, presents a certain number of limitations. A first important limit is the small number of DCD participants. It would have been valuable to include a larger sample of children with DCD to bring statistical power and to reinforce our results. However, this was not possible because of the difficulty in recruiting children with singular DCD, a rare diagnosis. This is the reason we chose to present here three case studies, whose different clinical profiles gave very interesting results as to graphomotor learning and transfer. In addition, single case studies are useful since they allow fine analyses both at a qualitative and quantitative level, an interesting point when considering heterogeneous disorders such as DCD. Although the results reported here cannot be generalized because of the high heterogeneity of DCD, this study nevertheless addresses a question that has rarely been investigated before, and our results pave the way to new research on this topic. Future research with larger sample sizes and taking into account the heterogeneity of the DCD will be necessary to confirm these results.

A second limit of this study is that participants were exposed to only one type of training. Future research should vary the training conditions, as the type of practice influences transfer [54,56]. Moreover, while we included two transfer tasks, it would be interesting to broaden the range of tasks along the near-to-far transfer continuum, including transfer to other pseudo-letters and to different handwriting styles (e.g., print versus cursive). The overarching aim is to determine which forms of training and transfer tasks are most effective or challenging for children with DCD, to better understand the development of their motor skills learning, and ultimately guide the development of targeted and efficient remediation programs.

## 5. Conclusions

These results contribute to a better understanding of DCD and its association with dysgraphia. Although the results need to be confirmed by studies involving a larger number of participants with different clinical profiles, this study highlights the fact that assessing graphomotor acquisition and transfer leads to different performances between children with DCD alone and children with DCD associated with dysgraphia. This, in turn, contributes to a better characterization of the disorder. Furthermore, these results highlight the importance of systematically assessing transfer when evaluating motor skill acquisition in these children.

## Figures and Tables

**Table 1:** Demographic characteristics of the TD children. MD = Manual Dexterity; A/C = Aiming And Catching; B = Balance.

n	Age	Gender (M/F)	Laterality (R/L)	Grade	MD	A/C	B	BHK Speed	BHK Qual
16	7.56 ± 0.24	7/9	14/2	2nd	11.06 ± 2.79	9.25 ± 2.57	10.37 ± 2.19	0.16 ± 0.74	0.73 ± 1.08
20	9.35 ± 0.28	10/10	18/2	4th	11.45 ± 2.80	9.80 ± 3.20	11 ± 0	−0.09 ± 0.67	0.79 ± 1.63
21	10.3 ± 0.25	12/9	18/3	5th	11.43 ± 2.65	9.24 ± 2.95	11 ± 0	0.09 ± 0.59	0.84 ± 0.58

**Table 2:** Demographic characteristics of the participants with DCD. MD = Manual Dexterity; A/C = Aiming And Catching; B = Balance.

	Age	Gender	Laterality	Grade	MD	A/C	B	Total (Percentile)	MABC-2 Checklist	BHK Speed	BHK Qual	Dysgraphia
DCD1	7.75	Male	R	2nd	14	4	5	7 (16)	7	0.40	0.61	No
DCD2	9.33	Male	R	4th	3	2	5	1 (0.1)	23	−1.65	−1.84	Yes
DCD3	10.83	Female	R	5th	8	6	6	6 (9)	7	0.54	−3.55	Yes

## Data Availability

The data presented in this study are available on request from the corresponding author due to ethical reasons.

## Abbreviations

The following abbreviations are used in this manuscript:

A/C	Aiming and Catching
B	Balance
APA	American Psychiatric Association
BHK	Brave Handwriting Kinder (Concise Evaluation Scale for Children’s Handwriting)
CI	Confidence Interval
DCD	Developmental Coordination Disorder
DSM	Diagnostic and Statistical Manual of Mental Disorders
LL	Lower Limit
MABC-2	Movement Assessment Battery for Children test
MD	Manual Dexterity
SNvpd	Signal-to-Noise Velocity Peak Difference
T1S1	Transfer 1 Session 1
T1S2	Transfer 1 Session 2
T2S1	Transfer 2 Session 1
T2S2	Transfer 2 Session 2
TD	Typically Developing
UL	Upper Limit
Zcc	Effect size for case–control studies

## Appendix A

**Table A1:** Pseudo-letter quality scale. Each item was scored 0 if the criterion was not present, and 1 or 2 (for the “global similarity to the model” criterion) if present. N.A.: not applicable.

Item	Score		
	0	1	2
Global similarity to the model			
One end touching the red dot			N.A.
No interrupted connection			N.A.
Starting from the red dot			N.A.
Correct proportion between the 2 guiding lines			N.A.
No retouching or erasure			N.A.
No strokes exceeding the guiding lines			N.A.
Respected angles			N.A.
Respected curves			N.A.
New segment quality (for combinations of pseudo-letters only)			N.A.

**Table A2:** Table summarizing the mean and standard deviation (SD) of the 7-year-old control group, the single values of the DCD1 child, and the *p*-values obtained for the comparison between the control group and the DCD1 child for the quality, the velocity, the SNvpd, and the number of abnormal stops. Zcc: Effect size for Case–Controls studies. 95% CI: 95% Confidence interval. LL: Lower Limit. UL: Upper Limit.

				Test	T1S1	T1S2	T2S1	T2S2	Diff T1S1-Test	Diff T1S2-Test	Diff T2S1-Test	Diff T2S2-Test
Quality	TD	**mean**		9.65	9.40	9.54	8.59	8.9	−0.26	−0.11	−1.06	−0.76
		**SD**		0.60	0.67	0.74	1.64	1.36	0.45	0.53	1.32	0.97
	DCD1	**score**		9.08	9.67	9.33	9.00	8.50	0.58	0.25	−0.08	−0.58
		***p***		0.19	0.35	0.39	0.41	0.39	0.045	0.26	0.24	0.43
		**Zcc**		−0.95	0.40	−0.28	0.25	−0.29	1.87	0.68	0.74	0.19
		**95%**	**LL**	−1.53	−0.12	−0.78	−0.25	−0.79	1.03	0.12	0.18	−0.31
		**CI**	**UL**	−0.34	0.91	0.22	0.74	0.22	2.68	1.22	1.29	0.68
Velocity	TD	**mean**		11.17	10.09	9.93	9.94	10.13	0.92	0.93	0.90	0.93
		**SD**		4.21	4.07	4.18	4.18	4.80	0.16	0.26	0.15	0.24
	DCD1	**score**		8.20	8.84	7.08	9.14	9.55	1.08	0.86	1.11	1.16
		***p***		0.25	0.38	0.26	0.43	0.45	0.17	0.40	0.10	0.18
		**Zcc**		−0.71	−0.31	−0.68	−0.19	−0.12	1.00	−0.27	1.40	0.96
		**95%**	**LL**	−1.25	−0.80	−1.22	−0.68	−0.61	0.38	−0.76	0.69	0.35
		**CI**	**UL**	−0.15	0.20	−0.12	0.31	0.38	1.59	0.23	2.08	1.54
SNvpd	TD	**mean**		14.53	17.30	16.53	37.90	34.69	2.77	2.00	23.36	20.16
		**SD**		5.65	7.63	5.35	14.87	11.65	5.03	4.57	11.38	10.12
	DCD1	**score**		15.50	11.67	15.17	18.83	17.00	−3.83	−0.33	3.33	1.83
		***p***		0.43	0.24	0.40	0.12	0.08	0.11	0.31	0.05	0.05
		**Zcc**		0.17	−0.74	−0.26	−1.28	−1.52	−1.31	−0.51	−1.76	−1.81
		**95%**	**LL**	−0.33	−1.28	−0.75	−1.94	−2.24	−1.98	−1.02	−2.54	−2.61
		**CI**	**UL**	0.66	−0.17	0.25	−0.60	−0.78	−0.63	0.02	−0.96	−0.99
Number	TD	**mean**		6.07	6.59	6.11	13.64	12.20	0.53	0.047	7.57	6.13
of stops		**SD**		3.52	3.62	2.63	6.03	5.69	2.25	2.96	3.70	4.57
	DCD1	**score**		5.17	5.33	5.67	9.17	9.00	0.17	0.50	4.00	3.83
		***p***		0.40	0.37	0.43	0.24	0.30	0.44	0.44	0.18	0.32
		**Zcc**		−0.26	−0.35	−0.17	−0.74	−0.56	−0.16	0.15	−0.97	−0.50
		**95%**	**LL**	−0.75	−0.85	−0.66	−1.29	−1.08	−0.65	−0.34	−1.55	−1.02
		**CI**	**UL**	0.25	0.16	0.33	−0.18	−0.02	0.34	0.64	−0.36	0.03

**Table A3:** Table summarizing the mean and standard deviation (SD) of the 9-year-old control group, the single values of the DCD2 child, and the *p*-values obtained for the comparison between the control group and the DCD2 child for the quality, the velocity, the SNvpd and the number of abnormal stops. Zcc: Effect size for Case–Controls studies. 95% CI: 95% Confidence interval. LL: Lower Limit. UL: Upper Limit.

				Test	T1S1	T1S2	T2S1	T2S2	Diff T1S1-Test	Diff T1S2- Test	Diff T2S1-Test	Diff T2S2-Test
Quality	TD	**mean**		9.89	9.85	9.88	9.92	9.69	−0.04	−0.008	0.026	−0.199
		**SD**		0.87	0.69	0.78	0.86	1.14	0.90	0.73	0.86	0.87
	DCD2	**score**		9.33	8.5	9.00	9.17	9.33	−0.83	−0.33	−0.17	0.00
		***p***		0.27	0.04	0.14	0.20	0.38	0.2	0.34	0.41	0.41
		**Zcc**		−0.64	−1.95	−1.14	−0.87	−0.31	−0.88	−0.44	−0.23	0.23
		**95%**	**LL**	−1.12	−2.70	−1.70	−1.38	−0.76	−1.39	−0.90	−0.67	−0.22
		**CI**	**UL**	−0.15	−1.19	−0.56	−0.34	0.14	−0.35	0.03	0.22	0.67
Velocity	TD	**mean**		10.37	9.36	8.67	8.64	8.81	0.91	0.83	0.85	0.86
		**SD**		3.52	3.29	3.39	2.88	3.13	0.17	0.14	0.12	0.16
	DCD2	**score**		7.73	7.34	6.23	6.58	7.26	0.94	0.79	0.84	0.93
		***p***		0.24	0.29	0.24	0.25	0.31	0.43	0.39	0.47	0.34
		**Zcc**		−0.72	−0.58	−0.72	−0.71	−0.49	0.18	−0.29	−0.03	0.44
		**95%**	**LL**	−1.21	−1.05	−1.21	−1.20	−0.95	−0.27	−0.73	−0.52	−0.03
		**CI**	**UL**	−0.22	−0.10	−0.22	−0.21	−0.02	0.62	0.17	0.36	0.89
SNvpd	TD	**mean**		14.78	17.97	18.71	40.65	39.71	3.19	3.93	25.87	24.93
		**SD**		4.56	7.08	6.53	11.97	12.77	4.85	4.82	9.01	10.77
	DCD2	**score**		13.17	20.83	23.33	36.17	30.50	7.67	10.17	23.00	17.33
		***p***		0.37	0.35	0.25	0.36	0.24	0.19	0.11	0.38	0.25
		**Zcc**		−0.35	0.41	0.71	−0.38	−0.72	0.92	1.30	−0.32	−0.71
		**95%**	**LL**	−0.80	−0.06	0.21	−0.82	−1.21	0.39	0.69	−0.76	−1.19
		**CI**	**UL**	0.10	0.86	1.19	0.08	−0.22	1.44	1.88	0.13	−0.21
Number	TD	**mean**		5.43	6.65	6.825	13.10	12.96	1.22	1.39	7.67	7.52
of stops		**SD**		1.96	2.87	2.45	4.40	3.87	2.14	1.87	3.35	3.08
	DCD2	**score**		5.67	8.50	9.67	14.67	13.83	2.83	4.00	9.00	8.17
		***p***		0.45	0.27	0.14	0.37	0.41	0.24	0.09	0.35	0.42
		**Zcc**		0.12	0.64	1.16	0.36	0.23	0.75	1.40	0.40	0.21
		**95%**	**LL**	−0.32	0.15	0.58	−0.10	−0.22	0.25	0.76	−0.06	−0.24
		**CI**	**UL**	0.56	1.12	1.72	0.80	0.67	1.24	2.01	0.85	0.65

**Table A4:** Table summarizing the mean and standard deviation (SD) of the 10-year-old control group, the single values of the DCD3 child, and the *p*-values obtained for the comparison between the control group and the DCD3 child for the quality, the velocity, the SNvpd and the number of abnormal stops. Zcc: Effect size for Case–Controls studies. 95% CI: 95% Confidence interval. LL: Lower Limit. UL: Upper Limit.

				Test	T1S1	T1S2	T2S1	T2S2	Diff T1S1- Test	Diff T1S2-Test	Diff T2S1- Test	Diff T2S2-Test
Quality	TD	**mean**		9.87	9.91	10.05	9.87	9.76	0.04	0.18	0.004	−0.11
		**SD**		0.63	0.48	0.53	0.97	0.76	0.56	0.72	0.91	0.87
	DCD3	**score**		5.75	6.17	6.33	6.00	6.17	0.42	0.58	0.25	0.42
		***p***		<0.001	<0.001	<0.001	<0.001	<0.001	0.26	0.30	0.40	0.28
		**Zcc**		−6.56	−7.87	−7.05	−3.97	−4.72	0.68	0.56	0.27	0.61
		**95%**	**LL**	−8.61	−10.32	−9.25	−5.26	−6.32	0.20	0.09	−0.17	0.14
		**CI**	**UL**	−4.50	−5.41	−4.84	−2.67	−3.21	1.15	1.01	0.70	1.07
Velocity	TD	**mean**		12.75	10.83	10.38	11.42	11.53	0.85	0.82	0.91	0.92
		**SD**		4.14	3.79	3.37	3.50	3.30	0.11	0.12	0.11	0.13
	DCD3	**score**		19.67	17.31	14.90	14.49	20.58	0.88	0.76	0.74	1.05
		***p***		0.05	0.05	0.10	0.20	0.007	0.40	0.32	0.07	0.17
		**Zcc**		1.67	1.71	1.34	0.88	2.74	0.27	−0.50	−1.55	1.00
		**95%**	**LL**	0.99	1.02	0.74	0.36	1.79	−0.17	−0.95	−2.18	0.46
		**CI**	**UL**	2.33	2.38	1.92	1.48	3.67	0.71	−0.04	−0.90	1.52
SNvpd	TD	**mean**		13.11	16.34	17.52	31.33	30.22	3.23	4.42	18.23	17.11
		**SD**		5.49	5.71	5.80	8.21	6.60	5.16	4.37	6.45	3.96
	DCD3	**score**		8.58	11.17	9.33	19.33	11.33	2.58	0.75	10.75	2.75
		***p***		0.17	0.19	0.09	0.08	0.005	0.45	0.21	0.13	0.001
		**Zcc**		−0.82	−0.91	−1.41	−1.46	−2.86	−0.13	−0.84	−1.16	−3.63
		**95%**	**LL**	−1.31	−1.41	−2.01	−2.07	−3.83	−0.55	−1.33	−1.71	−4.81
		**CI**	**UL**	−0.32	−0.39	−0.79	−0.83	−1.88	0.31	−0.33	−0.59	−2.43
Number	TD	**Mean**		4.25	5.33	6.13	10.22	10.19	1.08	1.87	5.97	5.94
of stops		**SD**		1.29	1.84	2.25	3.36	2.69	1.40	1.63	2.73	1.91
	DCD3	**Score**		4.25	3.33	4.50	9.50	5.67	−0.92	0.25	5.25	1.42
		***p***		0.50	0.15	0.24	0.42	0.05	0.09	0.17	0.4	0.016
		**Zcc**		−0.82	−0.91	−1.41	−1.46	−2.86	−0.13	−0.84	−1.16	−3.63
		**95%**	**LL**	−1.31	−1.41	−2.01	−2.07	−3.83	−0.55	−1.33	−1.71	−4.81
		**CI**	**UL**	−0.32	−0.39	−0.79	−0.83	−1.88	0.31	−0.33	−0.59	−2.43
